# A qualitative study of the vocational and psychological perceptions and issues of transdisciplinary nurses during the COVID-19 outbreak

**DOI:** 10.18632/aging.103533

**Published:** 2020-07-03

**Authors:** Jing Fan, Kaihui Hu, Xueqin Li, Ying Jiang, Xiang Zhou, Xin Gou, Xinyuan Li

**Affiliations:** 1 Department of Urology, The First Affiliated Hospital, Chongqing Medical University, Chongqing, China; 2 Department of Gynecology, The First Affiliated Hospital, Chongqing Medical University, Chongqing, China

**Keywords:** COVID-19, transdisciplinary nurses, interview, vocational problems, psychological issues

## Abstract

Background: Due to its high infectivity and concealment, the coronavirus disease 2019 (COVID-19) outbreak that occurred in Wuhan attracted global attention. A special nursing group of transdisciplinary nurses (TNs) who had not worked in respiratory medicine, infection departments, or emergency and intensive medicine but who accounted for a large proportion of all nurses also drew our attention. Few studies have examined this special group of TNs. Therefore, this study collected the experiences and views of TNs at the forefront of the COVID-19 outbreak to investigate their potential problems.

Results: Twenty-five TNs and 19 nurses with experience in infectious diseases (non-TNs) were enrolled in the study. Compared with non-TNs, TNs showed higher levels of perceived stress and relatively less perceived social support. For TNs, the ambiguous roles, transition of operating mode, unfamiliar work content, and reversal of their daily schedule were the most common vocational problems. Additionally, most TNs had psychological problems such as anxiety, pain and insomnia. The incomprehension of parents, concern for family members and long-term isolation were the most common causes of psychological stress.

Conclusion: This survey is the first to focus on the group of TNs at the forefront of the COVID-19 outbreak and to investigate their experiences, vocational issues and psychological stresses qualitatively and quantificationally. We found that TNs had more perceived stress and less perceived social support than non-TNs. The vocational and psychological issues of TNs should be highlighted. These findings identify important issues and offer insights into the underlying issues to help TNs ultimately win the battle against novel coronavirus epidemics.

Methods: Semi-structured and face-to-face individual interviews and quantitative assessments were conducted. The Braun Clarke Thematic Analysis method and the strategy outlined by Miles and Huberman were used in the data analysis process of the qualitative study. The perceived stress scale and perceived social support scale were utilized to quantificationally evaluate the perceived stress level and the amount of perceived social support. Both qualitative and quantitative methods were adopted to assess the vocational and psychological perceptions and issues.

## INTRODUCTION

In recent decades, there have been a variety of global coronavirus outbreaks, including severe acute respiratory syndrome coronavirus (SARS-COV) and Middle East respiratory syndrome coronavirus (MERS-COV), which have brought serious losses to human society [1, 2]. The coronavirus disease 2019 (COVID-19) outbreak, which occurred in Wuhan, HuBei Province, spread rapidly throughout the country and quickly attracted global attention [3, 4]. Given the high infectivity and concealment surrounding this outbreak, the government of China quickly generated containment strategies and performed a series of measures in the early stage of the outbreak. Throughout the defensive and therapeutic system, crucial roles were played by medical workers, a large proportion of whom were nurses. In this study, we employ semi-structured interviews in combination with a scale assessment to focus on a special group of nurses and investigate their experiences, issues and challenges during their frontline work against COVID-19.

As the “gatekeepers” of the health care system, nurses at the forefront of the COVID-19 outbreak played key roles in identifying suspected and confirmed COVID-19 patients by carefully evaluating disease manifestations and exposure history [5]. In addition, as “interrupters”, they implemented and maintained high-quality infection control measures to control the spread of COVID-19 [3, 5]. Because of the large-scale outbreak, multidisciplinary nurses from all over the country participated in epidemic prevention and control. A special nursing group of transdisciplinary nurses (TNs) who had not worked in respiratory medicine, infection departments, or emergency and intensive medicine but who accounted for a large proportion of all nurses attracted our attention [6].

A series of studies have highlighted the important roles of appropriate emotions and stress management, the satisfaction of basic needs, sufficient social support, clear task distribution and flexible working schedules on nurses’ work and psychological stress [7–9]. High-frequency and high-intensity work, including close contact with patients, produces occupational hazards and psychological stress for nurses. However, most researchers have placed more emphasis on nurses with experience in infectious diseases (non-TNs), especially those working in emergency and intensive medicine [10–12]. As a result, existing studies on TNs' experiences during the COVID-19 pandemic are limited, and quantitative and qualitative studies are lacking. Given TNs’ limited experience in nursing during infectious pandemics, we suspected that these nurses likely endured even greater vocational and psychological stress [13, 14]. Therefore, we designed the study to collect the experiences and views of TNs at the forefront of the COVID-19 outbreak and to evaluate their psychological stresses. The results will emphasize an important issue and offer insights into the underlying issues to help TNs ultimately win the battle against novel coronavirus epidemics. In the long run, these findings may help health care institutions prepare for future pandemics.

## RESULTS

In the present research, we primarily focused on the group of TNs at the front line against the COVID-19 outbreak and investigated their experiences, vocational issues and psychological stresses. In this part, the interrater reliability showed at least substantial agreement for every theme (Ƙ = 0.63-0.85).

### Awareness of nurses’ responsibilities and roles

When we asked the participants about the responsibilities and obligations of nurses in the face of sudden acute infectious diseases threatening public health, we heard many similar and unmistakable voices promoting “the Nightingale spirit”. One of the participants said,

“From the first day I became a nurse, I was deeply conscious of my responsibility to heal the wounded and rescue the dying. In the face of sudden novel coronaviruses, the lives and health of the world's population are under serious threat. As an angel in white, I have to summon “the Nightingale spirit” and go to the front lines where I am most needed to treat patients using professional knowledge and skills.”

In addition, all participating nurses described their roles in the COVID-19 outbreak. Most of them thought of themselves as nurses, friends or even family. They not only focused on physical fitness but also maintained the mental health of patients:

“First, I should assist doctors in treating patients and do my job well. Furthermore, most patients in isolation for a long time are bored, alone and scared. I should also be their friend and family to establish a harmonious and friendly relationship with them to help them maintain their mental health.”

When the participants mentioned changes in perceptions of nurses’ job responsibilities and obligations, some of them noted that they had obtained a more divine sense of purpose and would continue their future work with this sense of professional mission:

“In the past, I used to do my job well, stick to my post, or try to be a “five-star” nurse. But this outbreak has made me see that everyone has a responsibility in the face of the epidemic, and the numerous serious cases have made my sense of responsibility and mission stronger. Our essential work is to help patients alleviate pain. Facing great disaster, I should have great love!”

Other nurses thought that their increased experience and excellent professional skills; particularly techniques for dealing with acute respiratory infections, would be beneficial for further work:

“Although most nursing work in daily life is closely related to my primary major, mastering more comprehensive nursing skills is a better guarantee for the safety of patients. I can more skillfully and unhurriedly face the outbreak of other acute diseases endangering public health.”

### Recognition of responsibilities of transdisciplinary nursing work

We asked the participants, “With regard to respiratory infections, as a transdisciplinary nurse, how do you think the responsibilities differ from your usual work?” Differences in working contents and working patterns were the most common answers. One of the nurses in the surgical system said,

“Surgical work is based on ‘panic-mode’, and the faster work pace and turnover of patients is also significant. In addition to the routine work, I have to leave time to deal with some emergencies. However, the work mode here is mainly ‘process-based’, and the patient's condition is more complex; the disease is relatively continuous, and the work pace is also slower.”

Another nurse in another medical system said the work patterns were not exactly the same, with an obvious difference in the process of observing patients’ condition:

“For patients infected with COVID-19, I prefer to monitor the basic vital signs, such as temperature, blood pressure, respiration and oxygen saturation. I need to be constantly vigilant and accurately judge the changes in patients' conditions, especially the transition from mild to severe.”

We further investigated the challenges and problems produced by the transdisciplinary field in this epidemic prevention and control work, and we mainly heard three types of answers: acquiring new knowledge, enforcing new regulations and improving physical and psychological quality.

“Although I had learned nursing knowledge in different specialties before, with the update of knowledge and technology and in order to accurately treat patients, I need to relearn nursing knowledge about the COVID-19 outbreak. In addition, when facing large-scale respiratory infectious diseases, the work regulations are completely different from daily work, which also requires an adaptation process. Moreover, we went almost eight hours without eating and drinking in the isolation ward, so it is also a great test of physical quality and mental state. These [issues] were not encountered in my previous work.”

There is no doubt that there are many risks in nursing work, and the risks for transdisciplinary nurses are even greater in the fight against the COVID-19 outbreak.

“What I shouldn’t ignore is the risk of occupational exposure. Improper protection or careless operation will greatly increase the risk of infection. The unfamiliar operation of a protective suit can significantly increase the risk of infection. In addition, contradictions between patients and nurses remain. The increased workload and the adaptation to the new environment will lead to mental stress and physical fatigue; in this state, the quality of nursing work will also decline.”

When we mentioned the new understanding of nursing risks and risk prevention during frontline work against the epidemic, one of the TNs highlighted the importance of protection awareness and standardized operations:

“Acute infectious diseases are highly contagious and carry a high risk of infection, especially for health care workers who are in direct contact with patients. We must achieve accurate and standard operations, such as environment disinfection, detail control and protective suit operation. In addition, we should have a scientific understanding of the disease, enhance the awareness of protection, and successfully popularize knowledge. Ideological vigilance, attention to work, ensure mental health and physical health.”

### Psychological problems caused by transdisciplinary work

TNs play key roles in fighting at the forefront against the new coronavirus. However, when faced with acute respiratory infections, they have a relative lack of experience, and their psychological burdens increase remarkably under intense working pressure. Close social attention to their mental health is needed. When we asked about psychological problems when they were confronted with tough issues, worked in a strange and specific environment, faced high morbidity and mortality and worked under enormous pressure, most TNs answered that they experienced anxiety, grief, pain and insomnia:

“The professional preparation of disinfection, the intervention in patients' psychological problems and the document records were almost daily tasks, but I was not familiar and hadn’t been specifically trained. With the heavy workload every day, I sometimes felt anxious and had insomnia at night.”

“The high work intensity in the isolation ward, the disordered internal clock, and the restrictions and challenges of protective clothing all led me to be distressed.”

“Although facing life and death is common for nurses, I have never experienced so many deaths in the past. There was a growing body of critical patients dying while other new patients were constantly transferred to the intensive care unit every day. I often felt exhausted or even powerless. Not only was I sad that I could not treat my patients, but I also was sorrowful that I was not clear how long this situation would last.”

Given that the nursing areas are transdisciplinary, most of the participants’ families like did not understand why they had to be sent to the front. Therefore, the concerns of their families increased their psychological stress to some extent. Some nurses said they mainly worried about their children and parents:

“When I told my parents that I was going to the front, they didn't object, but it was clear they were worried and kept asking me why the TNs have to charge up. Although I explained patiently, I still worried about them.”

In some families, both members of a couple are medical workers and sign up to go to the front to treat patients. In addition to worrying about each other, they are concerned about their children's lives and safety:

“My husband is a doctor majoring in respiratory medicine, and facing the epidemic, he resolutely went to the front. Although I am a surgical nurse, I believe I can also make contributions to epidemic prevention and control. However, I still feel sorry and deeply miss my child.”

### The levels of perceived stress and perceived social supports

In addition, we conducted a quantitative comparison of the perceived stress levels and the amount of perceived social supports between TNs and non-TNs. The result of PSS showed TNs had the higher level of perceived stress, with the significantly higher perceived stress scores than non-TNs (9.88±2.12 vs. 2.58±3.65) (Supplementary Figure 2A and Table 1). Furthermore, in terms of the perceived social supports, TNs got the remarkably less scores of PSSS (71.72±3.29 vs. 78.68±2.45), which represented the lower level of the perceived social supports of TNs than non-TNs (Supplementary Figure 2B and Table 1).

**Table 1:** Results of PSS and PSSS.

**Group**	**N**	**PSS**		**PSSS**	
		Means±SD	95%CI	Means±SD	95%CI
**Non-TNs**	19	2.58±3.65	0.77-4.38	78.68±2.45	77.47-79.90
**TNs**	25	9.88±2.12	8.93-11.07	71.72±3.29	70.37-73.73
***t***	-	-7.585		7.556	
***P***	-	<0.001		<0.001	

All data were normally distributed.

CI: confidence interval, N: numbers, Non-TNs: nurses experienced in infectious diseases, PSS: perceived stress scale, PSSS: perceived social support scale, TNs: transdisciplinary nurses.

## DISCUSSION

This interview survey is the first to pay attention to TNs, who constitute a large proportion of all nurses at the front line against the COVID-19 outbreak, and to provide insight into their vocational and psychological issues caused by the transdisciplinary work. Based on a survey of 25 TNs and 19 non-TNs, higher perceived stress levels and less perceived social support were detected in the TN group. Following further interviews with TNs, we found that ambiguous roles, the transition of operating modes, unfamiliar work contents, the environment and intensity and the reversal of daily schedules were the most common vocational problems for TNs. Additionally, almost all of the TNs had psychological problems such as anxiety, grief, pain and insomnia. Unacceptable mortality and the resulting powerlessness, incomprehension of parents, concern for family members and long-term isolation were the most common causes of psychological stress. These findings are consistent with other studies investigating nurses in emergency departments [15]. However, TNs seem to suffer from more psychological stress.

From conventional nursing to risk-averse infection control, the transformation of responsibility is the first challenge for TNs [16]. Most TNs have never received training for acute respiratory infections, nor have they been exposed to similar tasks, such as environmental disinfection or special care paperwork. For example, the related high risk of infection among TNs is partly because they are trained to temporarily wear and remove protective equipment and are unfamiliar with their operation. Therefore, these nurses believe that improving the ability and experience of TNs and nursing students in epidemic prevention and control is necessary to face epidemic outbreaks. In addition, efficient and reasonable pre-job training is an effective way for TNs to more quickly adapt to epidemic prevention and control-related nursing work.

Many studies have shown that clear role recognition is an important prerequisite for better nursing work [17–19]. For example, Lam K. emphasized that detailed role classification was beneficial for improving work efficiency [20]. The present study showed that more than half of TNs play ambiguous roles, and most of them play the role of psychologists many times to assist patients with psychological disorders. To some extent, the ambiguous roles of TNs at the forefront of the epidemic resulted in vocational issues. The TNs in this study believed that although medical resources were scarce during the specific period of the outbreak of the new coronavirus, more detailed role classification, clearer role definitions and job descriptions, and appropriate suggestions for expanded responsibilities would be effective methods to alleviate role ambiguity and improve work efficiency.

An important but overlooked problem is the psychological issues of nurses on the front line of epidemics. Arnaud Duhoux [21] and Sarah K. Schäfer [22] summarized general mental health problems and posttraumatic stress symptoms as the two most common types of psychopathological issues among nurses. Unfavorable working hours, including long work weeks, night shifts, weekend work, and quick returns, severely affect biological rhythms and work-life balance [23]. Intensive job attributes, including long-term emergency situations and a fast working pace, lead to a constantly high-pressure state [15]. These job characteristics considerably increase the risk of general mental health problems, such as depression, anxiety, insomnia, pain, and grief [24]. Furthermore, the pandemic and the high number of sudden patient deaths can result in posttraumatic stress symptoms reflected in the four aspects of intrusion, avoidance, negative alterations in cognition and mood and alterations in arousal and reactivity [22]. Because of their long-term working experiences that involve intensive and specific work content on the front line of the epidemic, most non-TNs are more familiar with the situation and can readily accommodate it. In contrast, TNs who lack experience with this type of working schedule, environment and intensity on the front line have more difficulty adapting and consequently are more likely to develop psychological disorders.

Although the psychological stress of nurses has been demonstrated to be higher than that of other professions and although nursing is also a high-risk occupation for psychological disease, in the context of a large-scale epidemic of infectious diseases, more attention should be paid to the special group of TNs [25–27]. The present survey about the psychological stresses of TNs found that anxiety, pain, grief and insomnia were the most common psychological problems, which is similar to other nurse-related studies, but with different causes: (1) their colleagues may be infected, and the number of infected TNs is significantly higher than non-TNs, which leads to anxiety among the TNs; (2) TNs have not been in a closed working environment and worn protective suits for a long time, which greatly challenges their psychological and physical limits; (3) unfamiliar working modes and a lack of skill in the content of their work increase their psychological burden; (4) most TNs have difficulty accepting high mortality and helplessness in the face of the large number of severe patients; and (5) compared with non-TNs, TNs suffer from more pressure from their family, and combined with concerns about their family members, their psychological burdens are significantly increased.

The results of the study suggest that in addition to patients' mental disorders, more attention should be paid to the psychological health of nurses, especially TNs. We can establish a psychological consultation platform for medical workers and increase the rear security of front-line medical workers to reduce psychological pressure and maintain their mental health. Furthermore, entertainment and sports facilities, such as running and dancing, could be established, which would be helpful to adjust emotions and relieve pressure.

In the present study, we highlighted the existing issues of TNs at the front line of the COVID-19 outbreak and provided some insights to further address vocational problems and alleviate psychological stress. In subsequent work against pandemics, a more appropriate work schedule, effective pre-job training and more detailed role classification will ameliorate the related vocational issues. In addition, measurements such as psychological consultation platforms and entertainment and sports facilities should be provided to protect the psychological health of TNs.

The present results offer a new perspective on the group of TNs, evaluate the transdisciplinary deficiencies and address existing issues in the treatment of pandemic infectious diseases. However, some limitations remain to be further discussed. (1) Most enrolled TNs worked in the same hospital, which likely resulted in directivity caused by locality and reduced credibility and objectivity. (2) The sample size was relatively low, and a larger-scale survey might further enhance the practical value. In the future, more participants, including TNs and non-TNs from various hospitals, will be recruited to expand the study. (3) The quantifiable measurements are limited, and quantitative follow-up and assessments should be combined to more accurately identify the existing vocational and psychological issues caused by the transdisciplinary work and further improve the validity and quality of the research.

## CONCLUSION

This study aimed to investigate the existing vocational and psychological issues of TNs against the novel coronavirus and attempted to offer possibilities for this special nursing group. This is the first survey to focus on the group of TNs and to investigate their experiences and vocational and psychological problems during the COVID-19 outbreak. We found that TNs had higher perceived stress levels and less perceived social support. Ambiguous roles, unfamiliar work patterns, a lack of skill in the work content leading to higher infection rates among colleagues, and family factors are prominent problems. These findings provide important information and insights into the underlying issues to help TNs ultimately win the battle against novel coronavirus epidemics.

## MATERIALS AND METHODS

### Design

**Figure 1:**
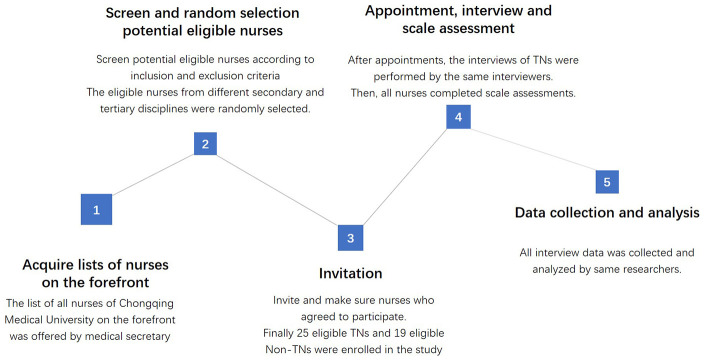
**The flowchart of the interview study.**

We conducted a qualitative study utilizing semi-structured and face-to-face interviews to investigate the experiences, vocational issues and psychological stresses of front-line nurses in the process of fighting against the COVID-19 outbreak. The qualitative descriptive method is usually employed to explore individual experiences, cognitions, and inclinations regarding a specific phenomenon [28]. The utilization of a qualitative descriptive method can promote understanding of the phenomenon by soliciting rich viewpoints and opinions from the perspective of participants [29]. Besides, the perceived stress scale (PSS) (Supplementary File 1) and perceived social support scale (PSSS) (Supplementary File 2) were employed to assess their perceived stress levels and the amount of perceived social support. PSS is a psychological instrument to measure nonspecific perceived stress, and PSSS contains seven-point Likert scale ranging from ‘strongly disagree’ to ‘strongly agree’. The flowchart of the entire study, from the screening of eligible nurses to data collection and analysis, is illustrated in Figure 1.

### Selection of participants

A purposeful sampling method was used in this study to recruit eligible participants. This sampling method is beneficial in helping researchers collect relevant and valuable information by identifying different participants [30]. In the selection of TNs, nurses were invited to participate in the study if they met the following criteria: (1) registered nurses who had not worked in respiratory medicine, infection departments, or emergency and intensive medicine; (2) nurses in the frontline hospital for COVID-19 in Hubei; (3) actively and directly provided care for patients; and (4) were willing to share their opinions and ideas. In contrast, eligible non-TNs were required to be registered nurses who had experience in respiratory medicine, infection departments, or emergency or intensive medicine. In addition, the non-TNs completed only some of the assessment scales. Because this study focused on the experience of front-line nurses in Hubei, nurses in management positions were excluded. Eligible individuals who were interested in participating in the study were contacted through email and were provided with detailed information on the research and the nature of their participation. Participants who were willing to participate in the study were asked to sign an informed consent form. Finally, 25 front-line TNs and 19 front-line non-TNs were enrolled in this study. Table 2 summarizes the demographic data of the participants. The demographic characteristics did not differ significantly between TNs and non-TNs. The various departments of the TNs and non-TNs are listed in Tables 3 and 4, respectively.

**Table 2:** Basic information.

**Items**	**TNs [n(%), n=25]**	**Non-TNs [n(%), n=19]**	**P values**
**Gender**			0.47
Female	21 (84)	17 (89.5)	
Male	4 (16)	2 (10.5)	
**Age (years)**			0.51
20-25	8 (32)	6 (31.6)	
26-30	9 (36)	8 (42.1)	
31-35	5 (20)	3 (15.8)	
36-40	2 (8)	2 (10.5)	
>40	1 (4)	0 (0)	
**Job title**			0.67
Nurse practitioner	20 (80)	15 (78.9)	
Supervisor nurse	5 (20)	4 (21.1)	
**Work experience (years)**			0.45
1-5	7 (28)	7 (36.8)	
6-10	11 (44)	8 (42.1)	
11-15	4 (16)	2 (10.5)	
>15	3 (12)	2 (10.5)	
**Marital status**			0.60
Single	6 (24)	4 (21.1)	
In love	2 (8)	2 (10.5)	
Ever-married	17 (68)	13 (68.4)	
**Childbearing history**			0.43
No children	9 (36)	8 (42.1)	
Be pregnant	0 (0)	0 (0)	
With children	16 (64)	11 (57.9)	

**Table 3:** Different departments of TNs.

**Departments**	**Results [n(%), n=25]**
**Surgical Department**	
General Surgery	5 (20)
Urology	2 (8)
Orthopedics	2 (8)
Neurosurgery	1 (4)
Cardiothoracic Surgery	1 (4)
**Internal Medicine**	
Vasculocardiology	2 (8)
Gastroenterology	2 (8)
Endocrine Medicine	2 (8)
Nephrology	1 (4)
Hematopathology	1 (4)
**Obstetrics and Gynaecology**	1 (4)
**Gerontology**	1 (4)
**Neurology**	1 (4)
**Dermatology and Venereology**	**1 (4)**
**Oncology**	1 (4)
**Otolaryngology**	1 (4)

**Table 4:** Different departments of Non-TNs.

**Departments**	**Results [n(%), n=19]**
Respiratory medicine	5 (26.3)
Infections department	4 (21.1)
Emergency and intensive medicine	10 (52.6)

### Ethical considerations

The research protocol was approved by the Ethics Committee of The First Affiliated Hospital of Chongqing Medical University. The study conformed to the ethical principles of medical research involving human subjects in the Helsinki Declaration [31]. The informed consent rights, privacy and anonymity of participants were protected.

### Data collection

**Figure 2:**
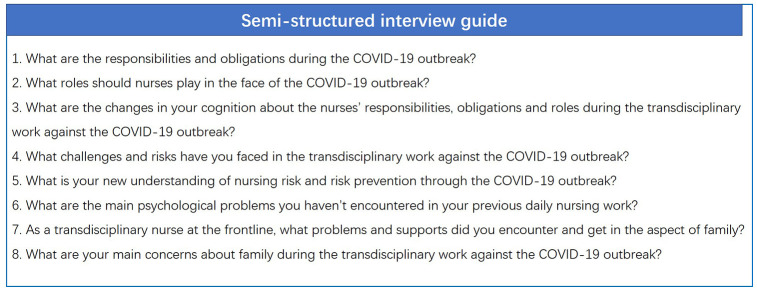
**The semi-structured interview guide.**

Semistructured face-to-face interviews with the participants were conducted by the first author to solicit their experiences, vocational issues and psychological states in the forefront of the COVID-19 outbreak. The interviews were arranged in a convenient place for the participants, such as the lounge. To facilitate the follow-up data analysis, all interviews were recorded and backed up with the permission of the participants. The participants were encouraged to express their views and opinions freely. An interview guide comprising open-ended questions was utilized to lead the conversations to the study areas [32] (Figure 2 and Supplementary Figure 1). The average time for an interview was 45 minutes, ranging from 30 to 60 minutes.

In the scale assessments, all participants completed the PSS and the PSSS. The PSS was the version reorganized by Mota-Cardoso et al. [33], consisting of 14 items with 5 alternatives per item, ranging from 0 to 4 points. The points indicated how often they felt or thought about certain events in the past month, from *never* (1 point) to *very often* (4 points). The internal consistency of PSS has been verified, with Cronbach's alpha = 0.90 in the study. The PSSS was composed of 12 items including the aspects of family, friend and others. Participants responded to the items on a 7-point scale representing the degree of agreement, form v*ery strongly disagree* (1 point) to *very strongly agree* (7 points). The internal consistency reliability coefficient of the PSSS was 0.91 in the present study. All participants completed the scales in a lounge and the whole process of filling in the two scales took around 40 minutes. Subsequently, three researchers simultaneously converted the results of the paper scales to the online version of the scales.

### Data analysis

Before the final data analysis of the interviews, the contents of each interview were recorded verbatim at the end of the day. All records were checked to ensure the accuracy of the transcription. Each record was analyzed within three days after the end of the corresponding interview. The Braun Clarke Thematic Analysis method and the strategy outlined by Miles and Huberman were used in the data analysis process [34, 35].

At the beginning of the data analysis process, all seven reliable researchers repeatedly read the interview records line by line and paragraph by paragraph to become familiar with the contents of the data. To develop preliminary codes, the narratives that were considered to be related to the phenomena in the study were emphasized. The records were scrutinized, and the relevant codes were further classified to form themes. Themes were also generated by codes that organized all of the data. We then reviewed these themes to refine the framework within and between themes to establish a network of themes and subthemes (Supplementary Table 1)**.**

The data dependability was established by checking codes among all researchers according to the strategy outlined by Miles and Huberman. All records were double coded by the research team. In all cases, we reached at least 80% agreement in assigning codes between two researchers. Disagreements were resolved by further discussion among the researchers. The investigator triangulation method enhanced the trustworthiness of the results.

Before analyzing the data of the PSS and PSSS, all paper scales were manually entered into the online scale version by three researchers simultaneously. When identical and credible results were obtained from the three researchers, the relevant data were further analyzed by SPSS (version 24). All quantitative data were normally distributed. MANOVAs, chi-square tests and t-tests for independent samples were employed to assess differences between non-TNs and TNs.

### Trustworthiness

Trustworthiness is the standard that constitutes the rigor of qualitative research [36]. The trustworthiness of this study was maintained by establishing four main standards, including credibility, confirmability, transferability and dependability. In terms of credibility, the content of the study was discussed through continuous communications between the researchers and the supervisor. The supervisor conducted a critical assessment to identify defects in the investigation and corrected them with the researchers. In terms of confirmability, member-checking with all participants was completed to validate the interpreted findings [37]. Participants were asked to verify the survey results to ensure that their opinions were accurately reflected in the data and to check the consistency between the results of the researchers and the actual intentions of the participants. In terms of transferability, we used a vivid description method to ensure sufficient and accurate contextual information. The findings and conclusions can be transferred to other studies with similar situations [38].

Dependability is achieved through the accurate records and the in-depth description of the methods used in the research. Besides, Cohen’s weighted kappa was employed to evaluate the interrater reliability. The poor agreement was considered if Ƙ < 0.00, slight agreement if between 0.00 and 0.20, fair agreement if between 0.21 and 0.40, moderate agreement of between 0.41 and 0.60, substantial agreement if between 0.61 and 0.80, and almost perfect agreement of between 0.81 and 1.00 [39].

## Supplementary Material

Supplementary Figures

Supplementary Table 1

Supplementary File 1

Supplementary File 2
