# Supplementary material for: A qualitative study of the vocational and psychological perceptions and issues of transdisciplinary nurses during the COVID-19 outbreak
Source: Aging (Albany NY). 2020 Jul 3;12(13):12479–92. doi: 10.18632/aging.103533 (PMC7377893; doi:10.18632/aging.103533)
Supplement: Supplementary Figures [file aging-12-103533-s004..pdf]

SUPPLEMENTARY FIGURES

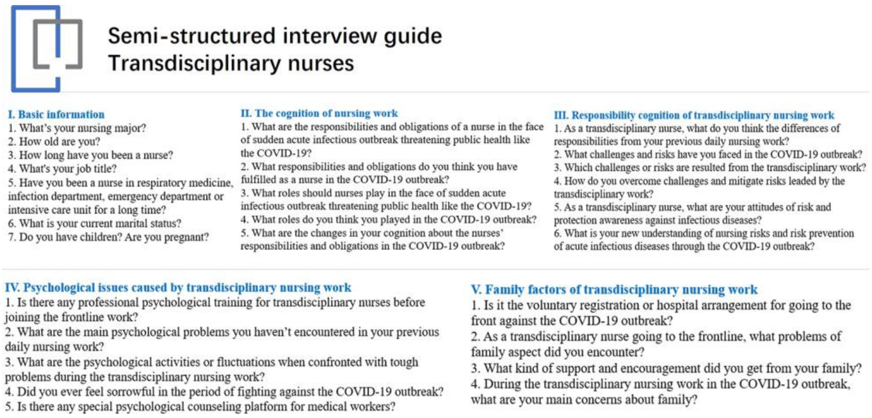

Supplementary Figure 1. The semi-structural interview guide including five parts.

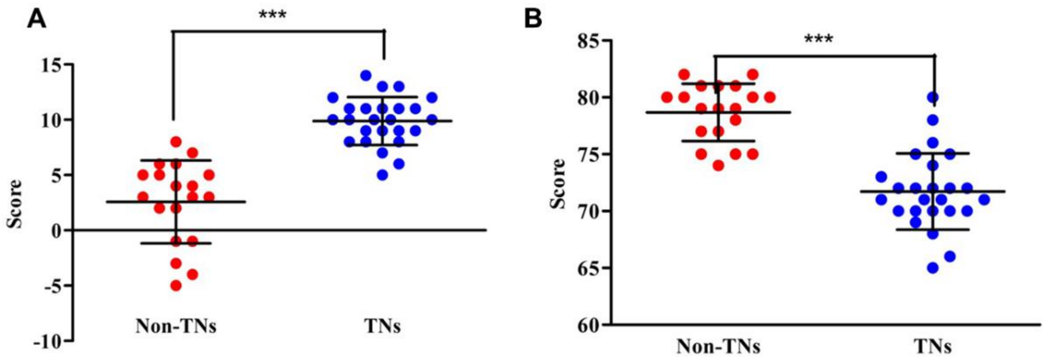

Supplementary Figure 2. The perceived stress levels and the amount of perceived social supports of TNs and Non-TNs. (**A**) the perceived stress scores, the higher score represents the higher level of perceived stress. (**B**) the perceived social support scores, the higher score represents the higher perceived social support level. The data are normally distributed, and are expressed as the means ± SD. ***$P <$ 0.001 TNs *vs.* Non-TNs.
