# Supplementary material for: A qualitative study of the vocational and psychological perceptions and issues of transdisciplinary nurses during the COVID-19 outbreak
Source: Aging (Albany NY). 2020 Jul 3;12(13):12479–92. doi: 10.18632/aging.103533 (PMC7377893; doi:10.18632/aging.103533)
Supplement: Supplementary Table 1 [file aging-12-103533-s003..pdf]

SUPPLEMENTARY TABLE

Supplementary Table 1. The data record table.

Themes and Sub-themes	Results [n (%), n=25]
**Responsibility cognition**	
healing the wounded and rescuing the dying	25 (100)
the Nightingale spirit	19 (76)
win the battle against the COVID-19	18 (72)
relieve mental and psychological pressure of patients	15 (60)
take care of the daily life of patients	7 (28)
**Role cognition**	
nurse	25 (100)
friend	22 (88)
family	17 (68)
psychotherapist	13 (52)
patient care	5 (20)
**New cognition of nursing work**	
**With new cognition**	25 (100)
stronger sense of responsibility and mission	21 (84)
more comprehensive nursing skills	20 (80)
playing a variety of different roles	16 (64)
full of love to patients and the job	13 (52)
**Without new cognition**	0 (0)
**Challenges of transdisciplinary nursing work**	
unfamiliar working patterns	25 (100)
unfamiliar working contents	23 (92)
standardized professional operations	20 (80)
occupational exposure and self-protection	20 (80)
physical and psychological quality	19 (76)
**Psychological issues**	
grief	22 (88)
insomnia	18 (72)
anxiety	16 (64)
pain	13 (52)
depressed	7 (28)
dysphoria	4 (16)
**Family factors of transdisciplinary nursing work**	
miss and worry about families	25 (100)
parents don't understand	12 (48)
spouse doesn't understand	8 (32)
guilt towards families	5 (20)
