# Supplementary material for: A qualitative study of the vocational and psychological perceptions and issues of transdisciplinary nurses during the COVID-19 outbreak
Source: Aging (Albany NY). 2020 Jul 3;12(13):12479–92. doi: 10.18632/aging.103533 (PMC7377893; doi:10.18632/aging.103533)
Supplement: Supplementary File 1 [file aging-12-103533-s002..docx]

**Perceived Stress Scale**

You will be asked to indicate how often you felt or thought about certain events in the past month. Although some of the questions are similar, there are differences between them and you should treat each one as a separate question. The best approach is to answer fairly quickly. That is, don’t try to count up the number of times you felt a particular way; rather indicate the alternative that seems like a reasonable estimate.

**The meaning of each number is as follows：**

**1 - never 2 - almost never 3 - sometimes 4 - fairly often 5 - very often**

| l. How often have you been upset because of something that happened unexpectedly? | 1 | 2 | 3 | 4 | 5 |
| --- | --- | --- | --- | --- | --- |
| 2. How often have you felt that you were unable to control the important things in your life? | 1 | 2 | 3 | 4 | 5 |
| 3. How often have you felt nervous and stressed? | 1 | 2 | 3 | 4 | 5 |
| 4. How often have you been able to handle the annoying things in your life? | 1 | 2 | 3 | 4 | 5 |
| 5. How often have you felt that you can effectively deal with important changes in life? | 1 | 2 | 3 | 4 | 5 |
| 6. How often have you felt confident about your ability to handle your personal problems? | 1 | 2 | 3 | 4 | 5 |
| 7. How often have you felt that things were going your way? | 1 | 2 | 3 | 4 | 5 |
| 8. How often have you found that you could not cope with all the things that you had to do? | 1 | 2 | 3 | 4 | 5 |
| 9. How often have you been able to control irritations in your life? | 1 | 2 | 3 | 4 | 5 |
| 10. How often have you felt that you can control things in your life? | 1 | 2 | 3 | 4 | 5 |
| 11. How often have you been angered because of things that happened that were outside of your control? | 1 | 2 | 3 | 4 | 5 |
| 12. How often have you been concerned about something that must be done? | 1 | 2 | 3 | 4 | 5 |
| 13. How often have you felt that you were able to control how to use your time? | 1 | 2 | 3 | 4 | 5 |
| 14. How often have you felt difficulties were piling up so high that you could not overcome them? | 1 | 2 | 3 | 4 | 5 |
