# Supplementary material for: A qualitative study of the vocational and psychological perceptions and issues of transdisciplinary nurses during the COVID-19 outbreak
Source: Aging (Albany NY). 2020 Jul 3;12(13):12479–92. doi: 10.18632/aging.103533 (PMC7377893; doi:10.18632/aging.103533)
Supplement: Supplementary File 2 [file aging-12-103533-s001..docx]

**Multidimensional Scale of Perceived Social Support**

Instructions: We are interested in how you feel about the following statements. Please read each statement carefully.

Circle the “1” if you **Very Strongly Disagree**

Circle the “2” if you **Strongly Disagree**

Circle the “3” if you **Mildly Disagree**

Circle the “4” if you are **Neutral**

Circle the “5” if you **Mildly Agree**

Circle the “6” if you **Strongly Agree**

Circle the “7” if you **Very Strongly Agree**

| 1.There is a special person (Leaders, relatives or classmates) who is around when I am in need. | 1 | 2 | 3 | 4 | 5 | 6 | 7 |
| --- | --- | --- | --- | --- | --- | --- | --- |
| 2.There is a special person (Leaders, relatives or classmates) with whom I can share joys and sorrows. | 1 | 2 | 3 | 4 | 5 | 6 | 7 |
| 3.My family really tries to help me. | 1 | 2 | 3 | 4 | 5 | 6 | 7 |
| 4.I get the emotional help & support I need from my family. | 1 | 2 | 3 | 4 | 5 | 6 | 7 |
| 5.I have a special person (Leaders, relatives or classmates) who is a real source of comfort to me. | 1 | 2 | 3 | 4 | 5 | 6 | 7 |
| 6.My friends really try to help me. | 1 | 2 | 3 | 4 | 5 | 6 | 7 |
| 7.I can count on my friends when things go wrong. | 1 | 2 | 3 | 4 | 5 | 6 | 7 |
| 8.I can talk about my problems with my family. | 1 | 2 | 3 | 4 | 5 | 6 | 7 |
| 9.I have friends with whom I can share my joys and sorrows. | 1 | 2 | 3 | 4 | 5 | 6 | 7 |
| 10.There is a special person (Leaders, relatives or classmates) in my life who cares about my feelings. | 1 | 2 | 3 | 4 | 5 | 6 | 7 |
| 11.My family is willing to help me make decisions. | 1 | 2 | 3 | 4 | 5 | 6 | 7 |
| 12.I can talk about my problems with my friends. | 1 | 2 | 3 | 4 | 5 | 6 | 7 |
